# Genetic Characterisation of the Bacterial Microbiota Associating With a Strain of *Epichloë* Fungal Endophyte of Perennial Ryegrass and the Interaction With Its *Paenibacillus* Members

**DOI:** 10.1111/1758-2229.70113

**Published:** 2025-06-08

**Authors:** Daniel A. Bastías, Linda J. Johnson, Sandeep Kumar, Ruy Jáuregui, Emma R. Applegate, Stuart D. Card

**Affiliations:** ^1^ AgResearch Limited Grasslands Research Centre Palmerston North New Zealand; ^2^ Animal Health Laboratory, Biosecurity New Zealand Ministry for Primary Industries Upper Hutt New Zealand; ^3^ The New Zealand Institute for Plant and Food Research Havelock North New Zealand

**Keywords:** antibiotic resistance, bacterial 16S rRNA gene, grasses, microbial networks, mutualism, population dynamics

## Abstract

Plant‐associated fungi can host unique bacterial microbiota to provide multiple benefits to their fungal hosts. Here it was characterised the bacterial microbiota associated with an *Epichloë* fungal endophyte (strain AR135) isolated from perennial ryegrass ( *Lolium perenne* ) via both 16S rRNA gene sequencing and direct microbial isolation and investigated the microbe‐microbe interactions between these bacteria and the fungus. The bacterial microbiota of AR135 was dominated by members within the genus *Paenibacillus*, with 99% of abundance (on average); although bacteria within genera *Delftia* and *Bradyrhizobium* were also present. *Paenibacillus* cells were located on the surface of hyphae of AR135 fungus in vitro on synthetic media and *in planta* within perennial ryegrass leaves. Two bacterial strains, E100 and E300, identified as *Paenibacillus*, were isolated from the AR135 mycelium. E300 drastically altered the abundance of both the whole bacterial microbiota (increased by 63%) and E100 (reduced to 0%). None of the variations observed in the abundance of total bacterial microbiota and E100 and E300 were associated with changes in the fungal biomass of *Epichloë*. The findings show that *Epichloë* fungal endophytes can host bacterial communities, the structure of which was regulated by key members of the bacterial community.

## Graphical abstract

The bacterial microbiota of *Epichloë* fungal endophyte AR135 was dominated by members within the genus *Paenibacillus*. Cells of *Paenibacillus* were located on the surface of AR135 fungal hyphae. E100 and E300 strains, identified as *Paenibacillus*, were isolated from the AR135 mycelium and showed a negative correlation when associated with AR135.

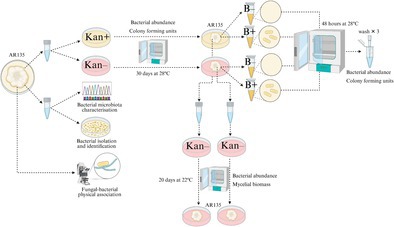

## 1 Introduction

Plants can benefit extensively by harbouring endophytic microorganisms, with the term endophyte defined as those microorganisms that spend all or part of their lifecycle within a plant, whereas causing no apparent disease symptoms (Collinge et al. 2022). Benefits include, but are not restricted to, plant growth promotion, increased seed yield, increased survival through the inhibition of phytopathogens and invertebrate pests, the removal of soil contaminants, improved tolerance to low fertility soils, and increased tolerance to extreme temperatures and drought (Card et al. 2016; Orozco‐Mosqueda and Santoyo 2021; Verma et al. 2022). As the study of endophytes intensifies, more complex plant–microbe and microbe–microbe interactions are identified. For example, it is now well documented that many species of bacteria can form symbiotic associations with plant‐associated fungi (including endophytes), and these associations can be classified according to the physical relationship with their host (ecto‐ vs. endosymbiotic), their level of symbiotic intimacy (obligate vs. facultative), their effect on plant fitness (promotional vs. inhibitory), and the ability of symbiotic bacteria to form biofilms (Bandara et al. 2006; Frey‐Klett et al. 2011; Bastías, Johnson, et al. 2020; Bastías et al. 2022; Duan et al. 2024). Bacterial ectosymbionts live on the outside of fungal hyphae or in close vicinity, whereas bacterial endosymbionts live within fungal hyphae (Bastías, Johnson, et al. 2020).

Plant‐associated fungi may be able to host unique bacterial microbiota that can provide multiple benefits to their fungal hosts (Kelliher et al. 2023; Duan et al. 2024). Most research on the microbiota of plant‐associated fungi has centred on arbuscular mycorrhizal fungi that commonly associate with bacterial ectosymbionts such as *Pseudomonas*, *Streptomyces*, and *Paenibacillus*, but also with endosymbionts including *Mycoplasma*‐related and *Burkholderia*‐related groups. Bacterial ectosymbionts of fungi largely feed on hyphal exudates that contain plant‐derived carbon, with these species generally exhibiting low levels of fitness dependency on their hosts, whereas bacterial endosymbionts heavily rely on the host for their nutritional needs and survival (Desirò et al. 2014; Duan et al. 2024). An example of an ectosymbiotic bacterium is *Pseudomonas mandelii* , a mycorrhizal helper, that can increase mycorrhizal colonisation within the host roots of *Helianthemum almeriense*, especially under drought stress (Guarnizo et al. 2023). Microbial communities can harbour keystone taxa which influence community structure and function via strong biotic interactions, independently of their abundances (Amit and Bashan 2023; Compant et al. 2025). Despite being common within plant microbial communities (Banerjee et al. 2018), few studies have identified keystone taxa within the bacterial microbiota of plant‐associated fungi (e.g., Jin et al. 2024).

Filamentous fungal endophytes of the genus *Epichloë* can form intimate associations with bacteria (Bastías, Jáuregui, et al. 2020; Bastías et al. 2023). *Epichloë* species have co‐evolved with cool‐season grasses of the sub‐family Pooideae with which they form long‐term symbiotic associations (Schardl et al. 2004; Bastías et al. 2024). *Epichloë* forms associations with perennial ryegrass ( *Lolium perenne* ), one of the most important forage species for ruminant production systems in temperate agricultural regions of the world, including New Zealand (Chapman et al. 2023). In natural and managed ecosystems, *Epichloë* can significantly impact fundamental ecological processes, species diversity, and food web structures (Bultman et al. 2012; Fuchs et al. 2013; Popay et al. 2021; Casas et al. 2022; Laihonen et al. 2022; Rasmussen et al. 2023). The prominence of *Epichloë* in many countries is owed to the fact that several species are used in agriculture as they confer bio‐protective properties to grass hosts, increasing pasture persistence and productivity with minor or complete absence of animal health issues (Kauppinen et al. 2016; Johnson et al. 2021; Card et al. 2024). Two ectosymbiotic bacterial strains, *Paenibacillus* sp. E222 and *Micrococcus luteus* E226, have so far been identified as associating with *Epichloë* within plants of perennial ryegrass (Bastías, Jáuregui, et al. 2020; Bastías et al. 2023). *Paenibacillus* sp. E222 has a rod‐shaped morphotype with potential to produce several bioactive secondary metabolites (Bastías, Jáuregui, et al. 2020) while *Micrococcus luteus* E226 has a coccoid morphotype and possesses plant growth promoting traits identified through whole genome analysis. When inoculated onto seeds of perennial ryegrass, already colonised by an *Epichloë* endophyte, E226 enhanced the production of leaves in the subsequent seedlings (Bastías et al. 2023). Analysis of the genome from E226 indicated that the bacterium can solubilise phosphate while producing vitamins and metabolic cofactors, which may explain the host plant's growth promotion. Furthermore, *M. luteus* E226 showed no impact on the mycelial biomass of *Epichloë in planta* or the production of bioactive *Epichloë*‐derived alkaloids responsible for invertebrate deterrence (Bastías et al. 2023).

We postulated that further tripartite associations, between perennial ryegrasses, *Epichloë* fungi and bacteria, exist and that these may have significant implications within an agricultural context (Duan et al. 2024). The objective was to genetically characterise the bacterial microbiota associated with cultures of *Epichloë* endophytes isolated from perennial ryegrass, and to isolate members of the bacterial microbiota to investigate the microbe‐microbe interaction between bacteria and fungal endophytes. Here it is reported the identification of the bacterial microbiota associated with *Epichloë* sp. strain AR135 via 16S rRNA gene sequencing and the isolation of two distinct bacteria (i.e., *Paenibacillus* spp. E100 and E300). Since both bacterial strains aligned within the same genus and had coexisted alongside the mycelia of *Epichloë in planta*, it is hypothesised that they would not be antagonistic to each other.

## 2 Material and Methods

### 2.1 Isolation of *Epichloë* sp. AR135

The fungal endophyte AR135 was isolated from several tillers originating from two plants of perennial ryegrass held in separate pots as part of an in situ fungal culture collection. In summary, the base of the tiller was dissected into small sections (2 mm) and surface disinfected by immersing them in 70% ethanol, followed by 10% sodium hypochlorite for 3 min before rinsing in sterile water (3 × 60 s). After drying within a sterile environment, the tissue sections were transferred to Petri dishes containing potato dextrose agar (PDA) without the addition of antibiotics and incubated at 22°C in the dark (Bastías, Jáuregui, et al. 2020). After approximately 2 weeks, *Epichloë* hyphae were observed emerging from the plant tissue sections. Five colonies of the fungus from each perennial ryegrass plant (10 colonies in total; 4–6 mm diameter) were sub‐cultured onto fresh PDA media.

### 2.2 Bacterial Microbiota Associated With AR135

**FIGURE 1:**
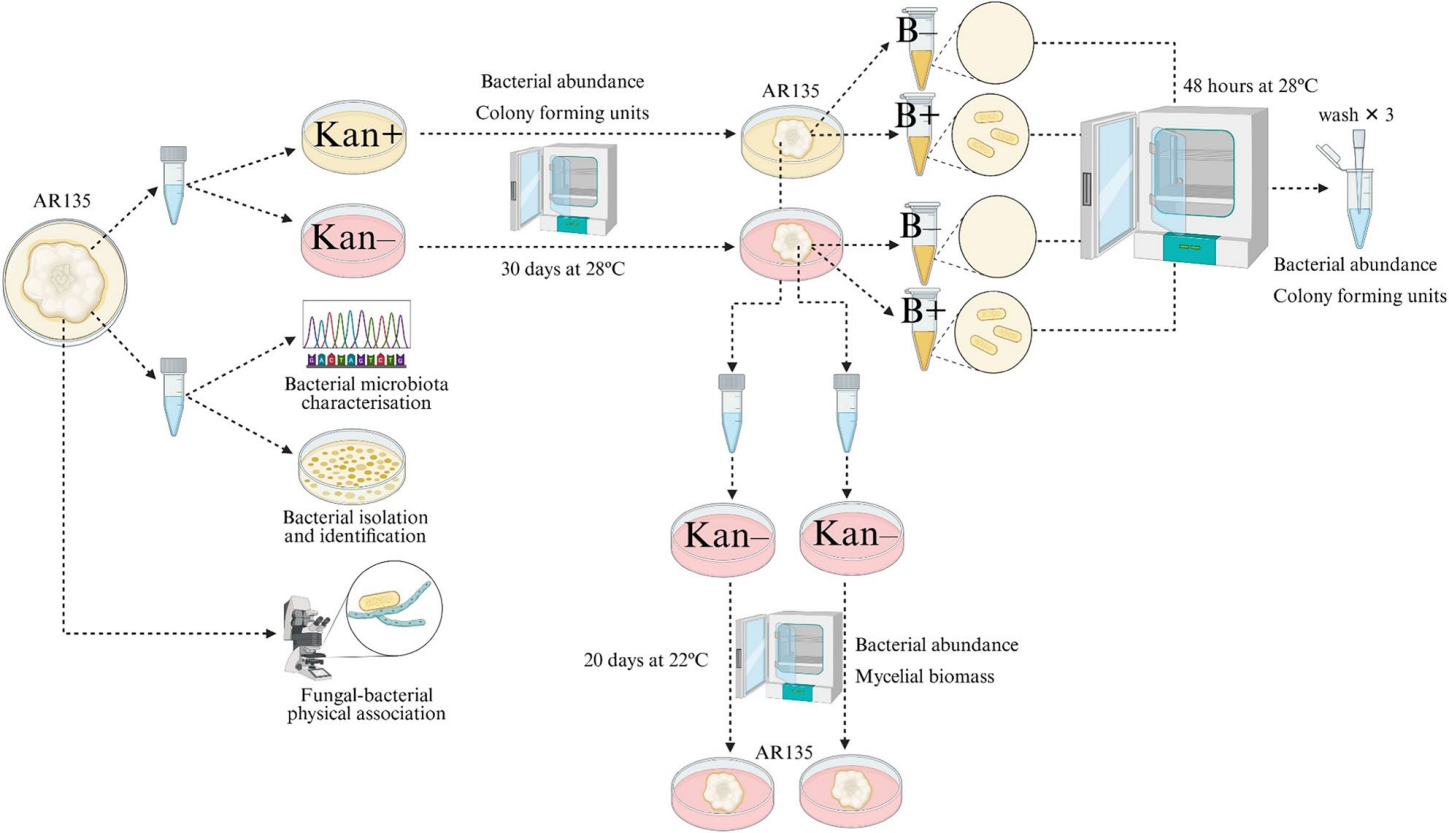
Summary of the experimental workflow since the isolation of *Epichloë* sp. AR135 from plants of perennial ryegrass. Mycelia of AR135 was treated with the antibiotic kanamycin (Kan+) or untreated (Kan−) and inoculated (B+) or non‐inoculated (B−) with *Paenibacillus* sp. E300. Some experimental steps were omitted for simplicity and details of the workflow are described in the main text. Created with BioRender.com.

The bacterial microbiota associated with AR135 was characterised by amplifying the V3‐V4 hypervariable region of the 16S rRNA gene with an Illumina MiSeq instrument (paired‐end, 2 × 300 bp) (Illumina Inc., USA) (Figure 1). *Epichloë* DNA was extracted from 80 to 100 mg of fresh fungal mycelia from eight out of the 10 AR135 colonies grown in PDA (four colonies from each perennial ryegrass plant, randomly selected). Prior to the DNA extraction, mycelial samples were homogenised in liquid nitrogen using a mortar and pestle within a sterile environment. DNA extraction was performed using the Quick‐DNA Fungal/Bacterial Miniprep Kit (Zymo Research Corp., USA) following the manufacturer's instructions. DNA integrity, quality, and quantity were determined using 1% agarose gels, a spectrophotometer (Nanodrop, Thermo Fisher Scientific Inc., USA), and Qubit 3 Fluorometer dsDNA BR Assay Kit (Invitrogen Corp., USA), respectively. Libraries for sequencing were prepared by Macrogen Inc. (Korea) using the primers 338F: 5′‐ACTCCTACGGGAGGCAGCAG‐3′ and 806R: 5′‐GGACTACHVGGGTWTCTAAT‐3′ (Wang, George, et al. 2024; Wang, Qu, et al. 2024).

#### 2.2.1 Processing of Sequencing Data

Paired‐end sequence data were processed using the Qiime 2 pipeline with modified parameters (Bolyen et al. 2019). Demultiplexed sequences were quality‐filtered using DADA2 (v1.14) to construct a feature table at the amplicon sequence variant (ASV) level (Callahan et al. 2016). Taxonomic assignments were performed using a Naïve Bayes classifier trained on the V3–V4 region of the SILVA (v138) and NCBI databases. The final dataset included 9 ASVs supported by 426,230 reads, with an average of 53,278 reads per sample (range 7772–82,068 reads) (see Table S1).

### 2.3 Isolation of Bacteria Associated With AR135

Fresh *Epichloë* mycelia was harvested from the 10 fungal colonies of AR135 grown in PDA and placed into a single 2 mL screw‐cap tube that contained sterilised glass beads and 1 mL of nutrient broth (NB; total fresh mycelium was 500 mg) (Figure 1). An additional tube containing glass beads and NB but without fungal mycelia was used as a control. The contents of all tubes were homogenised for 2 min at 2 m s^−1^ using the Bead Ruptor 24 (Omni International Inc., USA) and 2 μL of the supernatant from each tube was plated on nutrient agar (NA) and incubated at 28°C in the dark for 2 days. After incubation, two bacterial colony morphotypes emerged on the NA, whereas the control plate was free of bacterial colonies. The colonies were purified, and the two strains were identified by sequencing the bacterial 16S rRNA gene using the primers 27F and 1492R from DNA extracted with the Quick‐DNA Fungal/Bacterial Miniprep Kit from pure colonies (see Table S2) (Frank et al. 2008). The two strains were identified within the *Paenibacillus* genus (see result section) and designated E100 and E300.

### 2.4 Physical Association Between AR135 and *Paenibacillus* spp.

Fluorescence in situ hybridisation (FISH) was used to observe cells of *Paenibacillus* spp. associated with the hyphae of AR135 grown in vitro and associated with AR135 hyphae *in planta* (Bastías et al. 2023) (Figure 1). Bacterial cells were stained using probes EUB338 (5′‐GCTGCCTCCCGTAGGAGT‐3′) and BIF236 (5′‐GCCCATCCCCAAGTGACA‐3′) (labelled with cy3 and rhodamine green, respectively) that were specific for the domain eubacteria and genus *Paenibacillus* (Bertaux et al. 2005). The BIF236 probe resulted from a modification of the previously published BIF216 probe (Bertaux et al. 2003).

To reduce the density of *Epichloë* hyphae and therefore aid microscopic observation, AR135 subcultures were prepared by homogenising 1 mg of fresh fungal mycelia collected from the 10 colonies for 2 min at 2 m s^−1^ using the Bead Ruptor 24 and spreading a 20 μL aliquot of the subsequent solution across a Petri plate containing 4% water agar. After incubation at 22°C in the dark for 2 weeks, fungal mycelium was scraped from the media, transferred to a 2 mL tube and fixed with a 3:1 (v/v) solution of 3% paraformaldehyde and phosphate‐buffered saline (PBS). Plant tiller sections (2 mm in length), previously dissected for the isolation of *Epichloë* sp. AR135, and both bacterial strains (E100 and E300), were also fixed following the same protocol. Fungal, plant, and bacterial samples were then washed three times with PBS and immersed in 1:1 (v/v) solutions of PBS and 96% ethanol. A single sample consisting of a 1 mm^2^ square of fixed mycelium, plant tiller section, or 2 μL of fixed bacterial cells was mounted on hydrophobic Teflon‐coated slides within hybridisation wells (8 mm, Carl Roth GmbH & Co., Germany). Slides were dried overnight and dehydrated in 50%, 80%, and 96% ethanol. A 10 μL aliquot of filter‐sterilised lysozyme solution (10 mg mL^−1^; Serva, Global Science & Technology Inc., USA) was added to the samples, incubated at room temperature for 20 min, and pipetted off. Samples were immersed in 8 μL of hybridisation buffer (36 mM NaCl, 0.8 mM tris–HCl, 1.6% formamide, 0.0004% SDS, and sterile Milli‐Q water filled to 2 mL). A total of 1.5 μL of each bacterial probe (6.5 ng μL^−1^) was added to the hybridisation wells and a non‐EUB probe was also added as a binding control. Slides were incubated at 46°C for 2 h under high humidity in the dark and washed with wash buffer (460 mM NaCl, 20 mM tris–HCl, 5 mM EDTA, 0.01% SDS, and sterile Milli‐Q water filled to 50 mL). The slides were then prewarmed at 48°C, immersed in wash buffer, quickly dipped in icy water, and incubated at 39°C until dry. A 20 μL aliquot of 0.03% calcofluor white (Merck KGaA, Germany) was then added to the wells containing fungal and plant samples and all wells were filled with 8 μL Vectashield (Vector Laboratories Inc., USA). Fluorophore signals were detected with a FluoView‐FV10i confocal laser scanning microscope (Olympus Corp., Japan).

### 2.5 In Vitro Dynamics Between E100 and E300 on AR135 Cultures

The dynamics of E100 and E300 when co‐inoculated with mycelium of AR135 were studied in an experiment of exclusion and inoculation with E300. The experiment consisted of a 2 × 2 factorial design with the antibiotic kanamycin (Kan+, Kan−; 50 μg mL^−1^) and inoculation with the E300 bacterium (B+, B−) as main factors (Figure 1). The Kan treatment was used to exclude E300 (and not E100) from the AR135 mycelia. The E300 sensitivity and E100 resistance to Kan were determined with pure cultures of the bacteria (grown in NB for 48 h at 28°C in the dark). No colonies of E300 were detected on NA infused with the antibiotic (Kan+; 50 μg mL^−1^), whereas E100 colony numbers were similar between Kan+ and Kan− treatments on NA (380.66 ± 18.34 and 401.66 ± 6.93 colony forming units (CFU) per mL^−1^, respectively, after 48 h incubation at 28°C in the dark; *n* = 3).

A total of 500 mg of mycelia from the 10 colonies of AR135 were harvested and separately ground in 10 × 2 mL screw‐cap tubes as described previously. A 20 μL of supernatant from each of the 10 tubes was then spread across 10 separate Petri plates containing PDA infused with Kan (Kan+ plates) while a further 20 μL of supernatant from each tube was spread on 10 separate plates containing PDA without Kan (Kan– plates). After 30 days of incubation on Kan+ and Kan− plates, 200 mg of *Epichloë* mycelia was harvested from each plate and half of the Kan+ and Kan− produced samples were immersed in NB within 2 mL Eppendorf tubes enriched with E300 bacterium (B+; 15.50 CFU mL^−1^). The other half of the samples grown on Kan+ and Kan− were immersed in NB without E300 bacterium (B−). Both B+ and B− samples were incubated at 28°C for 48 h in the dark before the supernatant from the samples was removed, washed three times with sterile 0.1 M MgSO_4_ buffer, and their bacterial abundance assessed (see below for methods). Additionally, a further 200 mg of fungal mycelia was harvested from five Kan+ and five Kan− plates (randomly selected), ground, and the resulting supernatant spread across 10 separate PDA plates without Kan, as described previously. The subsequent biomass of AR135 mycelium was quantified after 20 days incubation at 22°C in the dark (Figure 1).

### 2.6 Quantification of Abundance of Total Bacterial Microbiota and Abundance of E100 and E300 Associated With the Mycelia of AR135

The abundance of total bacterial microbiota associated with AR135 was estimated by quantitative real‐time PCR (qPCR) (Figure 1). The abundance of E100 and E300 was determined by counting the number of bacterial CFUs that developed from the supernatant of ground AR135 total mycelia after incubation for 2 days in the dark on NA. Confirmation of the identity of E100 and E300 was undertaken by sequencing their 16 s rRNA gene regions and comparing this to the strain sequences held within our database. Both total bacterial abundance and strain‐specific bacterial abundance were quantified on AR135 mycelia cultured on PDA treated with Kan (for 15 days; Kan+, Kan−) or inoculated with E300 (from Kan plates; B+, B−), whereas total bacterial abundance was also quantified on AR135 mycelia (200 mg) transferred from PDA plates originally treated with Kan for 30 days to PDA plates free of Kan (for 20 days; Kan+ → Kan–, Kan− → Kan−) (Figure 1).

The abundance of total bacterial microbiota associated with AR135 mycelia was estimated as the number of bacterial 16S rRNA gene copies amplified with 27F and 1492R primers relative to the number of *Epichloë* nonribosomal peptide synthetase (NRPS1) gene copies amplified with forward 5′‐GTCCGATCATTCCAAGCTCGTT‐3′ and reverse 5′‐TGGTGGGAAGTTCCCTGCAG‐3′ primers (Castillo et al. 2006; Gagic et al. 2018). DNA was extracted from 100 mg of AR135 mycelia using the Quick‐DNA Fungal/Bacterial Miniprep Kit; reactions were prepared using the Kapa SYBR Fast kit (Biosystems Inc., USA), and DNA amplifications were undertaken in triplicate within 96‐well plates using a LightCycler 480 II (Roche Holding AG; Switzerland).

### 2.7 Statistical Analyses

The effects of Kan on total bacterial abundance and the abundance of E100 and E300 associated with the mycelia of AR135 were independently analysed with linear mixed effects models, using the function *lme* within the *nlme* package of R software (Pinheiro et al. 2009). The models included Kan (Kan+, Kan−) as the categorical factor and the individual plant as a random effect. The effects of inoculation of E300 on the abundance of total bacteria and specific bacterial strains (E100 and E300) associated with AR135 mycelia were independently analysed with linear mixed effects models using the function *lme*. The models included Kan (Kan+, Kan−) and E300 inoculation (B+, B−) as the categorical factors and the individual plant as a random effect. The relationship between the abundance of the bacterial strains E100 and E300 was analysed with linear mixed effects models, using the function *lme*. The model included the abundance of E300 as a continuous factor and the individual plant as a random effect. The effects of the removal of the antibiotic Kan on the abundance of the total bacteria associated with AR135 mycelia and the AR135 mycelial biomass were independently analysed also using the function *lme*. The models included the removal of the Kan antibiotic in AR135 mycelium previously treated and not treated with Kan (Kan+ → Kan−, Kan− → Kan−) as the categorical factor and the individual plant as a random effect. In all the analyses, normal distribution of errors was presumed, and model assumptions were met (residuals independence, normality, and variance homogeneity). For all variables, means ± standard errors of the mean (SEM) are reported.

## 3 Results

### 3.1 Bacterial Microbiota Associated With AR135, Identification of Bacteria Isolated From Mycelia of AR135, and Physical Association Between AR135 and Bacterial Strains

**FIGURE 2:**
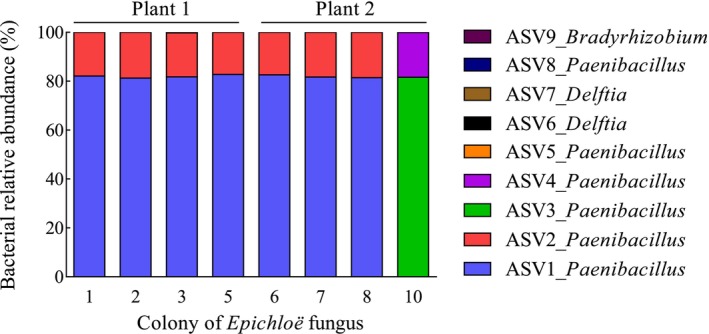
Relative abundance of the bacterial ASVs (amplicon sequencing variants) with 1 being the highest abundance and 9 being the lowest, associated with eight out of a total of 10 colonies of *Epichloë* sp. AR135 (colonies 4 and 9 were not quantified) originally isolated from tillers from two plants of perennial ryegrass. The database included 426,230 reads, with an average of 53,278 reads per sample (range 7,772–82,068 reads).

The bacterial microbiota associated with eight out of the 10 colonies of AR135, originally isolated from two perennial ryegrass plants, was composed of three genera, namely *Paenibacillus*, *Delftia*, and *Bradyrhizobium* (Figure 2). When compared with the NBCI database, the sequences associated with the *Paenibacillus* ASVs showed 100%–99.65% identity with an undetermined *Paenibacillus* species (i.e., *Paenibacillus* sp. strain LaBFR3109).

E100 and E300 were identified within the *Paenibacillus* genus by comparison of their 16S rRNA gene sequences with the NCBI database. E100 had 98.58% similarity with *Paenibacillus lautus* strain NBRC 15380 and E300 had 100% similarity with *Paenibacillus* sp. strain E222.

**FIGURE 3:**
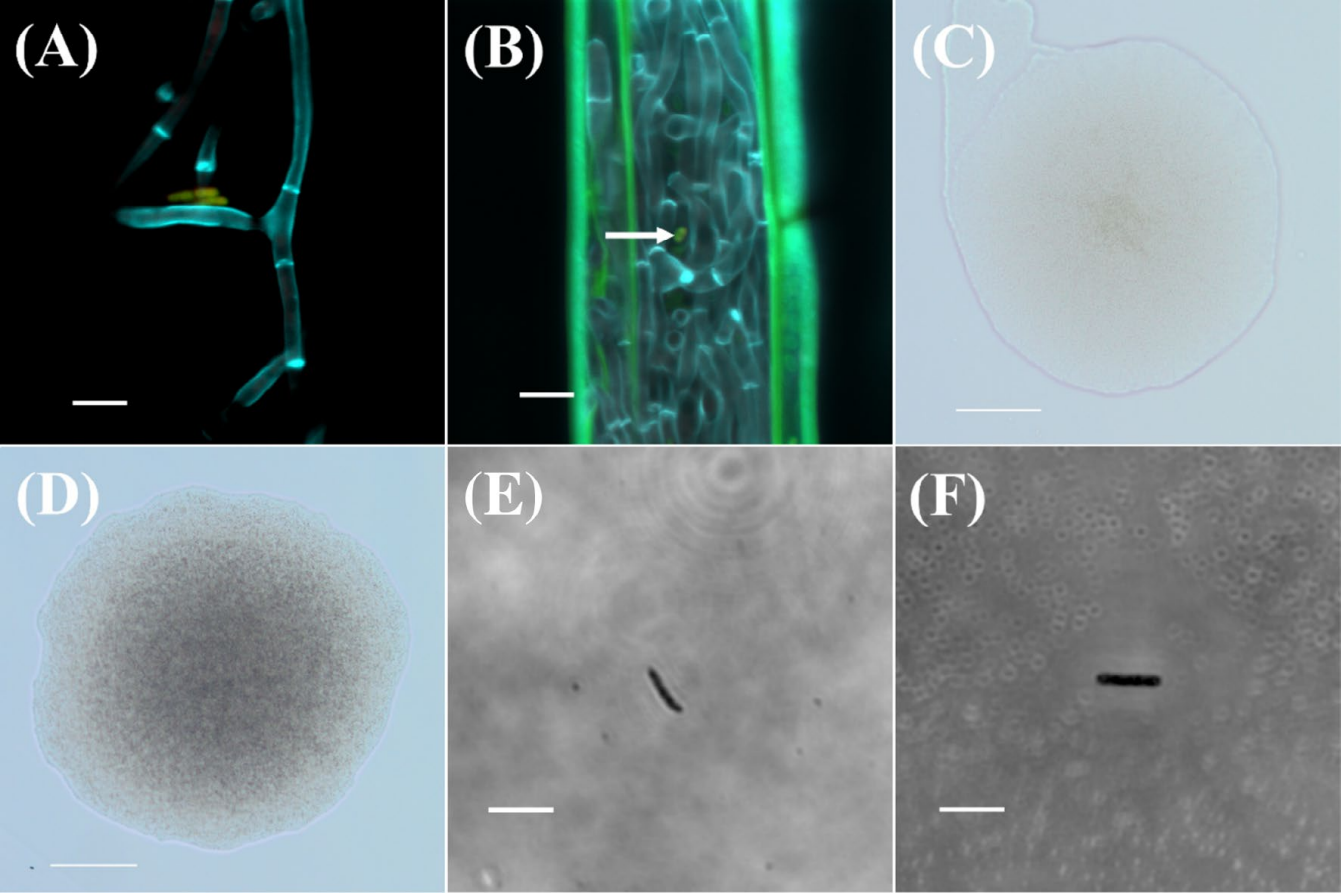
The in vitro physical association between hyphae of *Epichloë* sp. AR135 and cells of *Paenibacillus* spp. (A), the presence of *Paenibacillus* sp., alongside hyphae of *Epichloë* sp. AR135 *in planta* (B), colonies of *Paenibacillus* strain E100 (C) and E300 (D) isolated from ground hyphae of AR135, and cells of E100 (E) and E300 (F) in pure culture. *Paenibacillus* cells were labelled with bacterial domain‐specific EUB338 and genus‐specific BIF236 probes. Bacterial domain‐specific and genus‐specific probes were labelled with Cy3 (red) and Rhodamine green fluorophores, respectively and the superimposition of both fluorophore signals produced a yellow colour. Fungal cell walls were stained with calcofluor white (blue), and plant cells auto‐fluoresced green under the wavelength. Scale bars used in A, B, E, and F represent 5 μm while in C and D represent 100 μm. The arrow in B indicates a cell of *Paenibacillus* sp.

Cells of *Paenibacillus* spp., labelled with EUB338 and BIF236 probes, were located on the surface of hyphae belonging to AR135 in vitro when AR135 was grown on PDA (Figure 3A) and *in planta* within perennial ryegrass (Figure 3B). Colonies of E100 and E300 were approximately 420 μm in diameter after 48 h of incubation at 28°C in the dark, and showed limpid and pale morphotypes on NA, respectively (Figure 3C,D). Cells of both E100 and E300 were rod‐shaped, 4.3–4.9 μm in length with a 0.5–1.0 μm width (Figure 3E,F).

### 3.2 The Antibiotic and Inoculation Treatments Applied to AR135 Cultures Changed the Abundance of Total Bacteria and the Relative Abundances of E100 and E300

**FIGURE 4:**
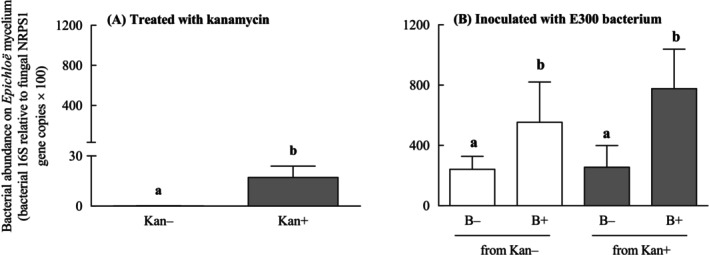
Abundance of total bacterial microbiota associated with the mycelia of *Epichloë* sp. AR135 treated with the antibiotic kanamycin (Kan+, shaded bars) or untreated (Kan−, unshaded bars) and inoculated (B+) or non‐inoculated (B−) with E300. Quantitative PCR (qPCR) was used to measure bacterial abundance relative to *Epichloë* NRPS1 gene copies from DNA extracted from mycelia treated with Kan for 15 days (A) and 48 h post inoculation with E300 (B). Different letters indicate significant differences at *p* < 0.05. Bars represent mean values ± standard errors of the mean (SEM) (*n* = 5).

The abundance of total bacteria associated with the mycelium of AR135 was dependent on the antibiotic Kan (*F*_(1,8)_ = 6.47, *p* = 0.034), increasing by 99% with the addition of the antibiotic to the media (Figure 4A). The abundance of the total bacterial microbiota associated with *Epichloë* mycelia as determined by qPCR varied with the inoculation of E300 independently of the previous Kan treatment (inoculation: *F*_(1,8)_ = 8.70, *p* = 0.018; Kan: *F*_(1,8)_ = 0.21, *p* = 0.653; inoculation × Kan: *F*_(1,8)_ = 1.05, *p* = 0.335). On average, the abundance of the total bacteria increased after the inoculation with E300 by 63% (Figure 4B).

**FIGURE 5:**
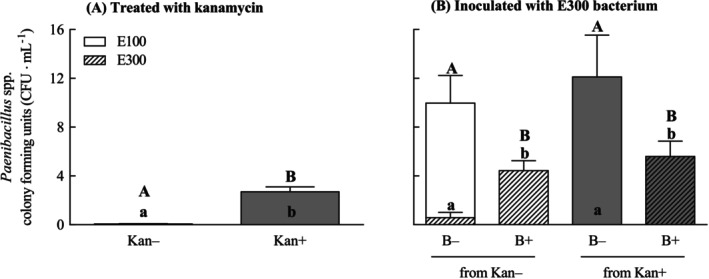
Colony forming units (CFU) of bacterial strains E100 (plain bars) and E300 (striped bars) originally isolated from the mycelia of *Epichloë* sp. AR135 treated (Kan+, shaded bars) and non‐treated (Kan−, unshaded bars) with the antibiotic kanamycin and inoculated (B+) and non‐inoculated (B−) with *Paenibacillus* sp. E300 bacterium. CFUs of E100 and E300 were independently measured alongside *Epichloë* mycelium treated with Kan for 15 days (A) and 48 h post inoculation with E300 (B). Uppercase and lowercase letters indicate CFU significant differences at *p* < 0.05 for E100 and E300 strains, respectively. Bars represent mean values ± standard errors of the mean (SEM) (*n* = 5).

**FIGURE 6:**
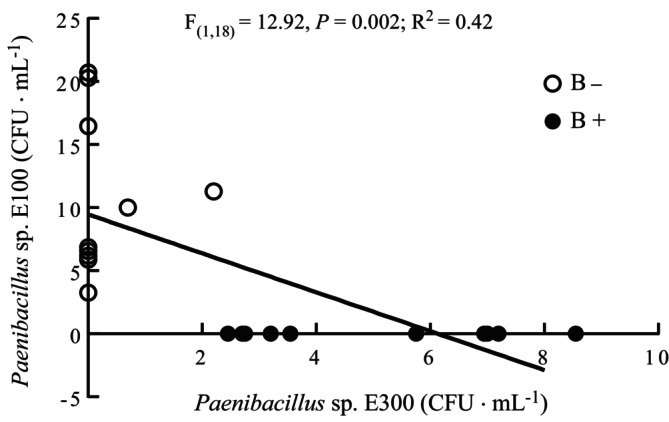
The relationship between colony forming units (CFUs) of bacterial strains E100 and E300 originally isolated from the mycelia of *Epichloë* sp. AR135 cultures inoculated (B+, black dots) and non‐inoculated (B−, white dots) with E300 bacterium for 48 h in nutrient broth. The abundance of E100 varied according to the abundance of E300 following the equation: E100 abundance (CFU mL^−1^) = 9.46–1.54 × E300 abundance (CFU mL^−1^) (*n* = 20).

The abundance of both E100 and E300 was dependent on the Kan treatment (E100: *F*_(1,8)_ = 44.44, *p* < 0.001; E300: *F*_(1,8)_ = 11.00, *p* = 0.011). As expected, treatment of AR135 mycelia with Kan resulted in the complete inhibition of E300 and no colonies of the bacterium were observed. Contrary to this result, the number of E100 colonies increased when the mycelia of AR135 were treated with Kan (Figure 5A). The abundance of E100 and E300 colonies varied when the mycelia of AR135 were inoculated with E300, and this result was independent of the previous Kan treatment (inoculation in E100 and E300: *F*_(1,8)_ = 27.34 and 45.75, *p* < 0.001; Kan in E100 and E300: *F*_(1,8)_ = 0.44 and 0.11, *p* = 0.523 and 0.741; inoculation × Kan in E100 and E300: *F*_(1,8)_ = 0.44 and 1.55, *p* = 0.523 and 0.247). As expected, the number of E300 colonies increased when the mycelia of AR135 were inoculated with E300 (i.e., this increase was *ca*. 3 fold, on average). The inoculation of the mycelia of AR135 with E300 negatively affected the abundance of E100, independently of the antibiotic treatment, with no CFUs of E100 identified (Figure 5B). There was a negative correlation between E100 and E300 that confirmed an antagonistic relationship between these two bacterial strains (Figure 6).

Sub‐culturing AR135 on antibiotic‐free media eased the Kan‐driven changes in the abundance of total bacterial microbiota and did not affect the mycelial biomass of the fungal endophyte.

**FIGURE 7:**
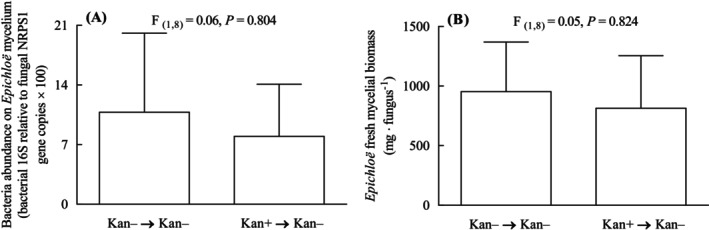
Abundance of total bacteria microbiota associated with the mycelium of *Epichloë* sp. AR135 (A) and mycelial biomass of AR135 cultures (B) grown on PDA that were treated or non‐treated with the antibiotic kanamycin (Kan) for 30 days and sub‐cultured and maintained on PDA free of antibiotics for 20 days (Kan+ → Kan− and Kan− → Kan−, respectively). Different letters indicate significant differences at *p* < 0.05. Bars represent mean values ± standard errors of the mean (SEM) (*n* = 5).

Despite the original Kan‐driven alteration in total bacterial abundance associated with the mycelium of AR135 (see Figure 3A), changes in total bacterial abundance were not detected on media where the antibiotic Kan was removed (Figure 7A). The mycelial biomass of AR135 also did not change on media where the antibiotic Kan was removed (Figure 7B).

## 4 Discussion

The bacterial microbiota associated with AR135 endophytic fungus, isolated from perennial ryegrass, was characterised via both 16S rRNA gene sequencing and direct microbial isolation. Although *Delftia* and *Bradyrhizobium* bacterial genera were identified in association with the mycelium of AR135, the genus *Paenibacillus* was the most abundant. Microscopical observations showed that *Paenibacillus* cells were located on the surface of hyphae of AR135 in vitro and *in planta*. Two strains of *Paenibacillus* spp., designated E100 and E300, were isolated from cultures of AR135. It was hypothesised that E100 and E300 would not show an antagonistic relationship with each other since they were both originally associated with *Epichloë* hyphae. Contrary to this, E300 negatively affected the abundance of E100 when both bacteria were co‐cultured alongside the mycelium of AR135. The variations observed in the abundance of total bacterial microbiota and E100 and E300 were not associated with changes in the fungal biomass of AR135 in vitro.

Fungi that form mutualistic symbioses with plants can harbour a unique bacterial microbiota. This microbiota is either located within or outside the fungal cells and is maintained by the fungal production of carbon‐derived metabolites and other compounds essential for bacterial survival (Desirò et al. 2014; Wang, George, et al. 2024). Here it is reported the first description of bacterial communities associated with cultures of *Epichloë* via 16S rRNA gene sequencing. *Paenibacillus* was the most abundant bacterial genus associated with the mycelia of AR135. *Paenibacillus* spp. have also been documented associating with other mutualistic fungi including mycorrhizae, showing growth promotional effects on the host fungus (Deveau et al. 2007). Cells of *Paenibacillus* were observed inhabiting the surface of fungal hyphae of AR135. This result agreed with experimental evidence from other plant beneficial fungi (i.e., mycorrhizal fungi) associated with *Paenibacillus* spp. that observed the bacterium alongside fungal hyphae (Toljander et al. 2006). *Paenibacillus* spp. and AR135 showed a similar physical association to that between *Epichloë* sp. AR60 and *Micrococcus* sp. E226 (Bastías et al. 2023). Since *Paenibacillus* is a key component of many plant‐associated microbiomes and associates with many grass species (Holl et al. 1988; Langendries and Goormachtig 2021; Li et al. 2021), it is likely that cells of the bacterium were isolated alongside mycelia of AR135 as the fungus emerged from surface disinfected, dissected, plant tissue (Muok and Briegel 2021). Bacterial genera *Delftia* and *Bradyrhizobium* were also found in association with some of the *Epichloë* cultures. Although further studies will need to be conducted in plant‐*Epichloë* associations, certain members within these bacterial genera have been described to promote the plant formation of symbioses with beneficial microbes (e.g., mycorrhizal fungi, rhizobia) (Morel et al. 2015; Sheteiwy et al. 2021).

The mycelia of AR135 were associated, at least, with two strains of *Paenibacillus* (i.e., E100 and E300). The enrichment of AR135 mycelia with *Paenibacillus* on artificial media could be explained by the fungal release of metabolites that *Paenibacillus* were capable of metabolising (although a media selection for particular bacterial groups cannot be ruled out) (Wang, George, et al. 2024; Wang, Qu, et al. 2024). Although this hypothesis needs to be evaluated, it has been reported that *Epichloë* exudates can promote the development of mycorrhizal fungi (Vignale et al. 2018). We speculate that *Paenibacillus* sp. E300 could be a keystone species within the bacterial microbiota associated with AR135, since the E300 addition/exclusion changed, to a great extent, the abundance of total bacteria and E100 (Banerjee et al. 2018). Interactions, including competition and antagonism, between bacteria, either between cells of the same species or between different bacterial species, are complex and ubiquitous in nature (Hibbing et al. 2010). The presence of E300 was found to decrease the abundance of E100. This antagonistic interaction was evident when artificial media infused with Kan specifically selected to inhibit E300 led to an increased abundance of E100 and when mycelium of AR135 inoculated with E300 resulted in a reduced E100 abundance. *Paenibacillus* are well known to produce antimicrobials against a broad range of bacteria (Wu et al. 2010; Grady et al. 2016). For example, the genome of E222 contained gene clusters coding for enzymes involved in the production of bacteriocins, lanthipeptides, type III polyketides, nonribosomal peptides, lasso peptides, siderophores, and terpenes (Bastías, Johson, et al. 2020). Bacteriocins are particularly relevant in the results of the present study since these compounds are normally toxic to bacteria closely related to the producing strain (recall that both E300 and E100 are *Paenibacillus* members) (Riley and Wertz 2002). Although not associated with the inhibitory effect of E300 on E100, it was remarkable the general increase in bacterial abundance that occurred in the inoculation treatment. This increase in the abundance of E100 may be related to the fact that after the inoculation event the mycelium of AR135 was immersed in a high nutrient medium (enriched and not enriched with E300) that likely promoted bacterial multiplication (Ohta and Hattori 1980).

The antibiotic Kan increased the abundance of total bacteria associated with the mycelium of AR135 (as determined by quantifying bacterial 16S gene copies), but this change in abundance was eased when the mycelium was sub‐cultured on PDA without antibiotics. This homogenisation in the bacterial abundance could be explained by a potential propagation of E300 (a putative keystone species), due to the absence of the antibiotic Kan in the medium, that negatively regulated growth of the members of the bacterial microbiota associated with *Epichloë* (Amit and Bashan 2023). Most importantly, the bacterial abundance documented in PDA plates free of antibiotics did not affect the amount of mycelial biomass of AR135. This later result suggests that, at least at the abundance level reported in the present study, the bacterial microbiota was not detrimental for the fungal growth, as documented in other plant beneficial fungi (Deveau et al. 2007; Battini et al. 2017). Further studies will need to determine if experimental or environmental conditions (e.g., high temperature and pH) that promote the propagation of the bacterial microbiota of *Epichloë* can lead to changes in the growth and bio‐protective properties of the fungus *in planta* (Frey‐Klett et al. 2011; Card et al. 2024).

## 5 Conclusions

The present study highlights that cultures of the endophyte AR135 grown on synthetic media presumed to be pure were instead co‐associated with distinct microflora, dominated by *Paenibacillus* spp. *Paenibacillus* cells were located on the surface of hyphae of AR135 in vitro on synthetic media and within leaves of perennial ryegrass. The *Paenibacillus* strains E100 and E300 were isolated from the mycelium of AR135. E300 largely affected both the whole bacterial microbiota and E100 in AR135 in the experiments of addition/exclusion of the bacterial strain. E100 and E300 showed an antagonistic relationship when the abundance of E300 was manipulated, whereas total bacterial microbiota abundance did not affect the amount of mycelial biomass when AR135 was grown in culture. The present study showed that similarly to other plant‐associated fungi (e.g., mycorrhizae), *Epichloë* endophytes were also associated with bacterial communities, and these communities contain presumably keystone taxa that shape their structures (Banerjee et al. 2018; Jin et al. 2024).

## Author Contributions

**Daniel A. Bastías:** writing – review and editing, writing – original draft, visualisation, validation, methodology, investigation, formal analysis, data curation, conceptualization. **Linda J. Johnson:** writing – review and editing, writing – original draft, resources, supervision, project administration, methodology, investigation, funding acquisition, conceptualization. **Sandeep Kumar:** writing – review and editing, writing – original draft, methodology, investigation, formal analysis. **Ruy Jáuregui:** writing – review and editing, writing – original draft, methodology, investigation, formal analysis. **Emma R. Applegate:** writing – review and editing, writing – original draft, methodology, investigation, formal analysis. **Stuart D. Card:** writing – review and editing, writing – original draft, resources, supervision, project administration, methodology, investigation, funding acquisition, conceptualization.

## Conflicts of Interest

The authors declare no conflicts of interest.

## Supporting information


**Table S1.** Matrix of bacterial ASVs (amplicon sequence variants) versus read counts associated with samples of *Epichloë* sp. AR135.

**Table S2.** 16S rRNA partial sequences of bacterial strains associated with *Epichloë* sp. AR135.

## Data Availability

The data that support the findings of this study are available in Table S1 and on request from the corresponding author.
